# Assessment of Anterior Segment Changes in Pseudophakic Eyes, Using Ultrasonic Biomicroscopic Imaging, after Pars Plana Vitrectomy with Silicone Oil or Gas Tamponade

**DOI:** 10.1155/2016/8303792

**Published:** 2016-05-19

**Authors:** Erkan Ünsal, Kadir Eltutar, Belma Karini, Osman Kızılay

**Affiliations:** Istanbul Research and Training Hospital, 34098 Istanbul, Turkey

## Abstract

*Objective*. To evaluate the morphological changes of the anterior segment using ultrasonic biomicroscopy (UBM) imaging in pseudophakic patients who underwent pars plana vitrectomy (PPV) with silicone oil or gas (C_3_F_8_) internal tamponade agent injection.* Method*. This prospective study included pseudophakic patients with planned PPV, divided into two groups according to internal tamponade agent: those in which silicone oil was used (*n* = 27, Group 1) and those in which gas (C_3_F_8_) was used (*n* = 24, Group 2). UBM measurements were performed in the supine position before and one week after surgery.* Results*. In patients of Group 1, postoperative trabecular meshwork-ciliary process distance (T-CPD) and iris-ciliary process distance (I-CPD), according to preoperative values, were found to be statistically significantly reduced, and postoperative mean value of scleral thickness (ST) and intraocular pressure (IOP), according to preoperative value, was found to be statistically significantly increased. In patients of Group 2, postoperative mean values of anterior chamber depth (ACD), ciliary body thickness (CBT), T-CPD, I-CPD, and IOP, according to preoperative values, were found to be statistically significantly reduced. Preoperatively, in Group 2 patients, according to Group 1 patients, TIA and IOP were found to be statistically significantly increased. Preoperative and postoperative IOP between the measured parameters with UBM showed no statistically significant correlation.* Conclusions*. Gases cause more morphological changes in the anterior segment structures. It is thought that complications such as increased intraocular pressure can be seen more frequently for this reason.

## 1. Introduction

Ultrasound biomicroscopy (UBM) technology uses high-frequency ultrasound to produce images of the anterior segment in high resolution. Anterior segment structures, including the crystalline lens, ciliary body, and lens zonules, can be morphologically assessed and quantitatively measured by using this in vivo noninvasive imaging technique. The reported repeatability of UBM measurements is good if the measurements are performed by the same experienced observer [1–4]. UBM has been investigated regarding the repeatability of measurements, accuracy, and precision [5–7]. Several publications have reported that intraobserver reproducibility is high for all the measurements performed using this technique, but interobserver reproducibility is poor [3, 7, 8]. The main reason for this variability is the quality of the received image. In addition, the variability in the measurement analysis should not be underestimated. The main reasons underlying this variability are the differences in the selection of the frame to be measured and the detection of the location of the scleral spur. Therefore, the comparison of the measurements before and after any attempt should be performed by the same observer. Thus, in our study, pre- and postoperative UBM measurements were performed and compared by a single observer.

In many vitreoretinal diseases, PPV is considered the standard surgical approach. Gases and silicone oil are used frequently as internal tamponade agents. Following PPV surgery, many complications can be encountered, a number of which are related to the anterior segment. UBM can reveal changes in the anterior segment due to complications in the postoperative period [9–16]. Among these changes are shallowing of the anterior chamber [9], narrowing of the anterior chamber angle, and ciliary body detachment [9, 11–13]. As a result of such changes, complications including glaucoma or hypotonia may occur [11].

The purpose of our study was to use UBM imaging techniques to analyze changes in the anterior segment morphology in the postoperative period of pseudophakic patients who had either PPV with silicone oil or PPV with gas (C_3_F_8_) injection.

## 2. Methods

Examination of the subjects' eyes was performed from October 2012 to March 2015, and 51 pseudophakic eyes of 51 patients with planned PPV were included in this prospective study. Patients were categorized as those who had silicone oil as an internal tamponade agent (Group 1) and those who had gas (C_3_F_8_) as an internal tamponade agent (Group 2).

The study was conducted in accordance with the tenets of the World Medical Association's Declaration of Helsinki. Approval of the local ethics of the study protocol was obtained from the Ethics Committee of the Istanbul Education and Research Hospital. All patients included in the study were informed about the details of the surgical procedure and signed an informed consent form.

One or two days before the surgery, all eyes underwent a complete ophthalmologic examination. A detailed history including age, gender, systemic diseases, and drugs used was recorded. The patients best-corrected vision acuity (BCVA) was measured using the Snellen chart. Goldmann Applanation Tonometry was used to measure intraocular pressure (IOP). The Goldmann three-mirror lens was used to evaluate the anterior chamber angle.

The following situations were excluded: patients with previous intraocular surgery (except cataract surgery); patients with ocular trauma history; cases of PPV that required associated surgical procedures, such as scleral buckling; previous anterior segment laser therapy; history of uveitis or glaucoma; use of any topical or systemic drug that might affect pupil or accommodation; and patients with postoperative intraocular pressure above 22 mmHg.

Indications for PPV included pseudophakic rhegmatogenous retinal detachments (RRD), pseudophakic diabetic retinopathy (DR) with persistent vitreous hemorrhage, pseudophakic fibrovascular proliferation, and pseudophakic tractional retinal detachment involving the macula, pseudophakic epiretinal membrane (ERM), and macular hole (MH).

Each patient underwent a 23-gauge pars plana vitrectomy by a single surgeon (EU). PPV was performed with a BIOM noncontact wide-angle viewing system. In patients with a retinal tear, we performed three to four rows of endolaser photocoagulation around the retinal tear using an endolaser probe. As a standard aspect of the surgery, we used twin light. After injection of triamcinolone acetonide, all cases were controlled for the presence of the posterior hyaloid membrane. In cases of an attached posterior hyaloid membrane, this was separated using the tip of a vitrectomy probe with vacuum, and, after that, it was surgically excised. In the appropriate cases, three to four rows of 360-degree endolaser photocoagulation were done prophylactically. Silicone oil (1000cs) was used as a long-acting internal tamponade agent; 14% C_3_F_8_ was used as short-acting internal tamponade agent. In all patients treated with silicone oil, sclerectomies were sutured. In cases treated with gas, sclerotomies were sutured only when needed. After the operation all patients were prescribed 1% prednisolone acetate and lomefloxacin drops in the form of one drop every two hours for a month.

UBM examinations were performed by the same surgeon, using the same device (SONOMED VuMAX II®) with a 35 mHz transducer. These examinations were performed before (24–48 hours) surgery and at one week (5–10 days) (7 ± 3 days) after surgery. In order to avoid the effects of drugs used such as paralysis of accommodation after atropine, cyclopentolate, or tropicamide use or mydriasis after phenylephrine use, all UBM measurements were performed after the effects of these drugs. Scanning was performed with the patient in the supine position. In order to use natural pupil dilation, scanning was performed in a room with low illumination. Accommodation was kept constant by asking the patient to stare at a red target on the ceiling.

After introducing topical 0.5% proparacaine HCL (Alcaine®, Alcon), a soft silicone eyecup of the appropriate diameter (18, 20, or 22 mm) was inserted between the upper lid and the fornix conjunctiva of the lower lid. Scanning of every patient before and after surgery was performed using the same eyecup. In order to prevent corneal contact, the focus distance of the transducer was set at 12 mm. For the purpose of immersion, the eyecup was filled with an adequate amount of sterile physiological saline.

Axial images of the anterior chamber and radial section of the angle images from the temporal quadrant were scanned. In order to obtain an ideal image and to have consistent pre- and postoperative measurements, we took care to have stable scanned axial images of the anterior segment (theoretically aligned with the central horizontal line and symmetrically to it) as well as stable images in the vertical alignment (cornea, lens, and anterior and posterior capsule should be balanced with the referenced vertical central line). When taking radial cross-sectional images of the angle, we made sure that the probe was perpendicular to the limbus of the scanned quadrant, and we chose the images with the best reflectivity of the iris [17]. In terms of accuracy and ease, localization of the scleral spur was given close attention in order to choose the images that best showed the ciliary body, the iris, and the reflectance of the interface between the ciliary body and the sclera.

**Figure 1:**
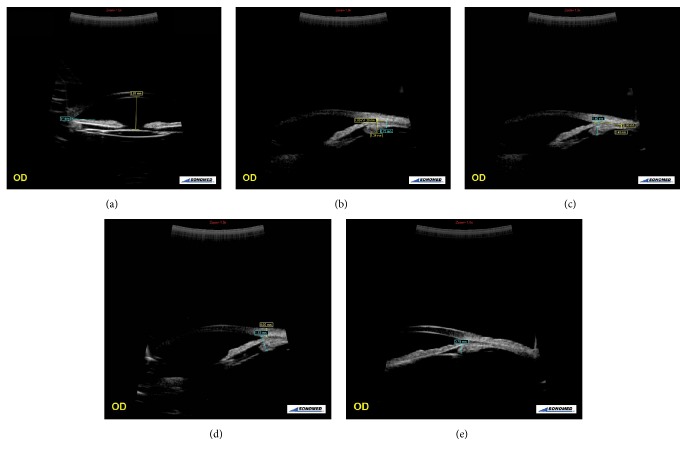
Views of axial images of the anterior chamber and radial section of the angle images from the temporal quadrant. (a) An UBM image of anterior chamber depth (ACT) and trabecular meshwork-iris angle (TIA). (b) An UBM image of ciliary body thicknesses 1 and 2 (CBT 1 and 2). (c) An UBM image of ciliary body thickness 3 (CBT 3) and the maximum ciliary body thickness (CBT Max). (d) An UBM image of scleral thickness (ST) and trabecular-ciliary process distance (T-CPD). (e) An UBM image of iris-ciliary process distance (I-CPD).

The anterior chamber depth and lens thickness were measured from the axial images of the anterior segment, using the methods previously recommended by Pavlin et al. and the scales provided in the user's guide for the device [18, 19]:

1. Axial ACD measurement: detected by measuring the distance between the posterior surface of the central cornea and the anterior surface of the IOL in the midline of the pupil (Figure 1(a)).
2. Trabecular meshwork-iris angle (TIA): measured with the apex in the iris recess and the arms of the angle passing through a point on the trabecular meshwork 500 *μ*m from the scleral spur and a point on the iris perpendicularly opposite (Figure 1(a)).
3. Ciliary body thickness (CBT) measured in four regions:
  - the distance 1 mm from the scleral spur (CBT 1), detected by measuring the distance between the posterior surface of the sclera and ciliary body to the border perpendicular to the surface of the sclera (Figure 1(b));
  - the distance 2 mm from the scleral spur (CBT 2), detected by measuring the distance between the posterior surface of the sclera and ciliary body to the border perpendicular to the surface of the sclera (Figure 1(b));
  - the distance 3 mm from the scleral spur (CBT 3), detected by measuring the distance between the posterior surface of the sclera and ciliary body to the border perpendicular to the surface of the sclera (Figure 1(c));
  - the thickest location of the ciliary body (CBT Max), detected by measuring the distance between the posterior surface of the sclera and ciliary body to the border perpendicular to the surface of the sclera (Figure 1(c)).
4. Scleral thickness (ST): the distance of the episcleral surface measured perpendicular to the scleral spur (Figure 1(d)).
5. Trabecular meshwork-ciliary process distance (T-CPD): measured as a line extending from a point 500 *μ*m anterior to the scleral spur along the corneal endothelium and dropped perpendicularly through the iris to the most anterior ciliary process seen during scanning in that meridian (Figure 1(d)).
6. Iris-ciliary process distance (I-CPD): the distance measured between the iris pigment epithelium and ciliary processes (Figure 1(e)).

One week (5–10 days) after the surgery, IOP was measured and we repeated at the same meridian (temporal quadrant) all the UBM examinations. We compared the values determined before and after the surgery. We used SPSS 15.00 for Windows software (SPSS Inc., Chicago, Illinois, USA) to evaluate the findings of this study. Comparison of the parameters of the same group was done using the paired *t*-test. The comparison of independent groups was done using the independent samples *t*-test. Pearson's correlation analysis was used to compare IOP and measured parameters with UBM. The *P* values of <0.05 were accepted as being statistically significant.

## 3. Results

Despite postoperative antiglaucomatous drugs, five patients with IOP more than 22 mmHg were excluded due to the fact that the initiative surgery of lowering IOP may have been needed.

This prospective study included pseudophakic patients with planned PPV, divided into two groups according to internal tamponade agent: those in which silicone oil was used (*n* = 27, Group 1) and those in which gas (C_3_F_8_) was used (*n* = 24, Group 2).

Patient characteristics by group are included in Table 1. Distribution of the patients diagnoses is shown in Table 2.

In our study, it appears that there are marked differences in the diagnosis distribution between the groups. Group 1 patients are mainly composed of RRD and PD patients, while Group 2 consists primarily of ERM patients.

The comparisons of the preoperative and postoperative parameters of the anterior segment and the angles of the temporal quadrant and IOP are shown, respectively, in Table 3.

In patients of Group 1, postoperative T-CPD and I-CPD, according to preoperative values, were found to be statistically significantly reduced, and the postoperative mean value of Stand IOP according to preoperative value was found to be statistically significantly increased (Table 3).

In patients of Group 2, postoperative mean values of ACD, CBT 1, CBT 2, CBT 3, CBT Max, T-CPD, I-CPD, and IOP according to preoperative values were found to be statistically significantly reduced (Table 3).

**Figure 2:**
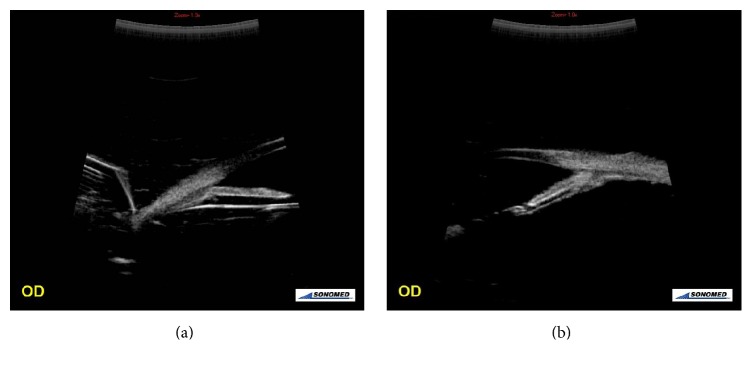
Views of radial section of the angle images from the temporal quadrant. (a) UBM image of an eye with silicone oil used as internal tamponade agent. (b) UBM image of an eye with gas (C_3_F_8_) used as an internal tamponade agent.

Comparison of the between-groups preoperative and postoperative parameters of the anterior segment and the angle of the temporal quadrant and IOP are shown, respectively, in Table 4. Preoperatively, in Group 2 patients, according to Group 1 patients, TIA and IOP were found to be statistically significantly increased. Postoperatively, in Group 2 patients, according to Group 1 patients, TIA was found to be statistically significantly increased, and CBT 2, CBT 3, and IOP were found to be statistically significantly decreased (Table 4, Figure 2). Preoperative and postoperative IOP between the measured parameters with UBM were not statistically significantly correlated (*P* > 0.005, *r* < 0.25).

## 4. Discussion

ST was found to be increased in the group of patients in which silicone oil was used as an internal tamponade agent. In Group 2 patients of our study, postoperative mean values of ciliary body thickness, T-CPD, and I-CPD, according to preoperative values, were found to be statistically significantly reduced.

Marigo et al. [20] in a study of 20 patients reported no statistically significant differences in the morphology of the anterior segment measurements performed with UBM preoperatively and one month after vitrectomy. In contrast to our study, Marigo did not use an internal tamponade. Our study consists of two groups: a group in which silicone oil was used and another group in which gas (C_3_F_8_) was used as internal tamponade agents. In addition, our study aimed to show early postoperative morphological changes of the anterior segment, so we performed the postoperative measurements one week after the surgery in order to show the effects of gas as an internal agent.

Çalik et al. [21] evaluated anterior segment changes with Pentacam Scheimpflug camera pre- and postoperatively in patients who underwent pars plana vitrectomy (PPV) and silicone oil injection. They reported that there was no change in TIA values of PPV patients with or without internal tamponade of silicone. Similarly, in our study, there was no change in TIA values of PPV patients with internal tamponade of both silicone oil (Group 1) and gas (Group 2).

Kim and Yu [22] evaluated the ciliary body thickness using UBM pre- and postoperatively in patients who underwent PPV for diffuse DME. They reported that the average preoperative value of CBT in DME patients was found to be thicker than the control groups. The CBT decreased significantly after PPV in DME group. This decrease in CBT correlates with the decrease of ciliary body edema. In our study, the decrease in CBT in patients with gas tamponade unlike those with silicone tamponade was considered a result of the pressure that gases exert on ciliary body because of surface tension rather than the ciliary body edema reduction.

Neudorfer et al. [14] evaluated the short-term changes induced by PPV on anterior segment morphology by means of high-frequency UBM. In the study patients were classified according to the gas tamponade usage. They found a significant decrease in ACD in the eyes of patients who had undergone gas tamponade but not in those who had not undergone gas tamponade. Similarly, we found a significant decrease in ACD in the eyes of patients who had undergone gas tamponade (Group 2). However, we did not find a significant decrease in ACD in the eyes of patients who had undergone silicone oil tamponade (Group 1). In the same study [14] no correlation was found in increase of IOP and ACD. In our study, cases with IOP above 22 mmHg after the operation were not considered because they were excluded from the study.

In patients in Group 2, postoperative mean values of ACD, CBT 1, CBT 2, CBT 3, CBT Max, T-CPD, and I-CPD, according to preoperative values, were found to be statistically significantly reduced. We concluded that there was much more pressure on the ciliary body in patients who were given gas in the supine position compared to the silicone-treated patients. This may be due to the fact that the surface tension of gases is higher than the surface tension of silicone oil and may be related to the spherical structure of silicone oil inside the eye which exerts less pressure on the ciliary body.

The mean value of ST of Group 1 patients was statistically significantly higher than the mean value of ST of Group 2 patients because, in patients treated with silicone oil, all access sites were closed by scleral sutures, which may result in episcleral edema. But the access sites of patients treated with gas tamponade (Group 2) were sutured only when needed.

After the first week of operations changes in the anterior segment have been described in the posterior segment, such as vitrectomy and scleral buckling [9–12, 23–25]. However, there is limited information on the effects of vitrectomy on the morphology of the anterior segment. If we could detect the effects of vitrectomy on the morphology of the anterior segment, we would be able to prevent complications in the predisposing eyes.

PPV with silicone oil and with gas as an internal tamponade agent causes changes in the parameters of the anterior segment. It seems that a significant number of postoperative complications arise due to the changes of these parameters. A better understanding of the changes in these parameters can be important to prevent such complications.

Hasegawa et al. compared IOP elevations in the immediate postoperative period after vitrectomy for various vitreoretinal disorders and to determine the incidence of and risk factor for IOP elevation [26]. They found that the IOP five hours after surgery was significantly lower in patients with MH than in those with DME, PDR, PVR, or RRD. The IOP one day after surgery was significantly lower in patients with MH and ERM than in those with DME, PDR, or RRD. In the same study, IOP elevation (>25 mmHg) was found in approximately one-quarter of cases within one day. In our study, despite postoperative antiglaucomatous drugs, five patients (9.8%) with IOP more than 22 mmHg after the operation were excluded.

In terms of the shortcomings of our study, besides the early postoperative (one week) UBM measurements, late postoperative measurements were not performed. However, the reduction of the amount of intraocular gas used as an internal tamponade agent (C_3_F_8_) combined with the advancing time causes postoperative decreased activity. This departs from the aim of the study. Although no significant correlation exists between IOP and the measured parameters with UBM, another limitation is the exclusion of eyes with postoperative IOP above 22 mmHg. In addition our study only includes a small sample size of each disorder, which may have influenced morphological changes of the anterior segment among vitreoretinal disorders. Intraobserver reproducibility is high for all the measurements performed using this technique, but interobserver reproducibility is poor [27]. Therefore, UBM examinations were performed by the same surgeon. On the other hand, this condition may have caused bias which can be one of the limitations of our study. Besides the quality of the image acquisition and the analysis differences, UBM evaluation of the anterior chamber may be affected and influenced by physiological and environmental variables. Factors such as room illumination, fixation, and accommodative efforts of the patient can cause several changes in the anterior segment anatomy. Thus, these factors should remain constant when dealing with quantitative measurements. In our study all measurements where performed according to a protocol with standard environment and conditions.

**Figure 3:**
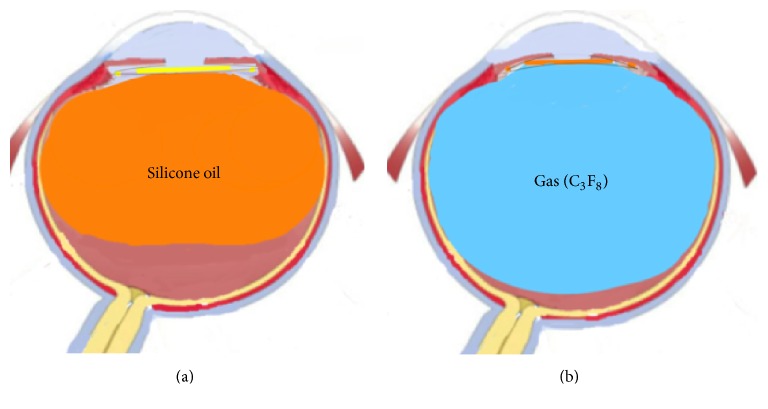
Views of demonstrative image: (a) silicone oil internal tamponade; (b) gas (C_3_F_8_) internal tamponade.

Gases do cause more morphological changes in the anterior segment structures (Figure 3). It is thought that complications such as increased intraocular pressure can be seen more frequently for this reason. Future studies with a large sample size and using more advanced devices will further facilitate our understanding of the morphological changes of the anterior segment in patients undergoing vitrectomy.

## Figures and Tables

**Table 1:** Demographic and clinical characteristics of the groups.

	Group 1 (*n* = 27)	Group 2 (*n* = 24)
Mean age ± SD (range)	60.3 ± 11.3 (41–77)	69.25 ± 9.8 (56–84)
Male/female	15/12	6/18
Eyes affected, *n* (right/left)	15/12	6/18
IOP (preoperative) mmHg, mean ± SD (range)	11.68 ± 2.86 (7–18)	14.10 ± 2.30 (10–18)
IOP (postoperative) mmHg, mean ± SD (range)	18.92 ± 2.23 (14–22)	12.81 ± 2.74 (9–19)

IOP: intraocular pressure; SD: standard deviation.

**Table 2:** Distribution of the patients diagnoses.

	Group 1 (*n* = 27)	Group 2 (*n* = 24)	Total
PDR	6	3	9
RRD	18	3	31
ERM	No	13	13
MH	No	2	2
IVH	3	3	6

PDR: proliferative diabetic retinopathy; RRD: rhegmatogenous retinal detachment; ERM: epiretinal membrane; MH: macular hole; IVH: intravitreal hemorrhage.

**Table 3:** Comparison of the preoperative and postoperative parameters of the anterior segment and the angle of the temporal quadrant.

	Group 1 (*n* = 27)			Group 2 (*n* = 24)		
	Pre	Post	*P* ^*∗*^	Pre	Post	*P* ^*∗*^
ACD, mean ± SD (mm)	3.37 ± 0.76	3.36 ± 0.67	0.914	3.55 ± 0.39	3.29 ± 0.40	**0.000**
TIA (°), mean ± SD	24.77 ± 9.6	27.44 ± 7.2	0.156	33.12 ± 8.74	31.37 ± 5.99	0.124
CBT 1, mean ± SD (mm)	1.11 ± 0.23	1.07 ± 0.18	0.076	1.13 ± 0.21	1.05 ± 0.25	**0.000**
CBT 2, mean ± SD (mm)	0.72 ± 0.22	0.71 ± 0.23	0.654	0.67 ± 0.23	0.59 ± 0.17	**0.000**
CBT 3, mean ± SD (mm)	0.55 ± 0.19	0.58 ± 0.24	0.251	0.50 ± 0.14	0.35 ± 0.09	**0.047**
CBT Max, mean ± SD (mm)	1.28 ± 0.19	1.23 ± 0.21	0.077	1.23 ± 0.24	1.13 ± 0.22	**0.000**
ST, mean ± SD (mm)	0,94 ± 0.08	1.02 ± 0.09	**0.004**	0.94 ± 0.07	0.97 ± 0.14	0.113
T-CPD, mean ± SD (mm)	1.43 ± 0.19	1.34 ± 0.12	**0.014**	1.41 ± 0.14	1.31 ± 0.11	**0.000**
I-CPD, mean ± SD (mm)	0.95 ± 0.17	0.87 ± 0.16	**0.002**	0.96 ± 0.19	0.84 ± 0.12	**0.030**
IOP, mean ± SD (range) (mmHg)	11.68 ± 2.86 (7–18)	18.92 ± 2.23 (14–22)	**0.000**	14.10 ± 2.30 (10–18)	12.81 ± 2.74 (9–19)	**0.002**

ACD: anterior chamber depth; LT: lens thickness; TIA: trabecular meshwork-iris angle; CBT 1, 2, and 3: ciliary body thicknesses 1, 2, and 3 mm, CBT Max: maximum ciliary body thickness; ST: sclera thickness; T-CPD: trabecular meshwork-ciliary process distance; I-CPD: iris-ciliary process distance; SD: standard deviation; ^*∗*^paired *t*-test.

**Table 4:** Comparison of the between-groups preoperative and postoperative parameters of the anterior segment and the angle of the temporal quadrant and IOP.

	Pre			Post		
	Group 1	Group 2	*P* ^*∗*^	Group 1	Group 2	*P* ^*∗*^
ACD, mean ± SD (mm)	3.37 ± 0.76	3.55 ± 0.39	0.310	3.36 ± 0.67	3.29 ± 0.40	0.681
TIA (°), mean ± SD	24.77 ± 9.6	33.12 ± 8.74	**0.002**	27.44 ± 7.2	31.37 ± 5.99	**0.041**
CBT 1, mean ± SD (mm)	1.11 ± 0.23	1.13 ± 0.21	0.695	1.07 ± 0.18	1.05 ± 0.25	0.767
CBT 2, mean ± SD (mm)	0.72 ± 0.22	0.67 ± 0.23	0.481	0.71 ± 0.23	0.59 ± 0.17	**0.039**
CBT 3, mean ± SD (mm)	0.55 ± 0.19	0.50 ± 0.14	0.106	0.58 ± 0.24	0.35 ± 0.09	**0.000**
CBT Max, mean ± SD (mm)	1.28 ± 0.19	1.23 ± 0.24	0.482	1.23 ± 0.21	1.13 ± 0.22	0.126
ST, mean ± SD (mm)	0,94 ± 0.08	0.94 ± 0.07	0.948	1.02 ± 0.09	0.97 ± 0.14	0.094
T-CPD, mean ± SD (mm)	1.43 ± 0.19	1.41 ± 0.14	0.762	1.34 ± 0.12	1.31 ± 0.11	0.349
I-CPD, mean ± SD (mm)	0.95 ± 0.17	0.96 ± 0.19	0.862	0.87 ± 0.16	0.84 ± 0.12	0.528
IOP, mean ± SD (range) (mmHg)	11.68 ± 2.86 (7–18)	14.10 ± 2.30 (10–18)	**0.002**	18.92 ± 2.23 (14–22)	12.81 ± 2.74 (9–19)	**0.000**

ACD: anterior chamber depth; LT: lens thickness; TIA: trabecular meshwork-iris angle; CBT 1, 2, and 3: ciliary body thicknesses 1, 2, and 3 mm, CBT Max: maximum ciliary body thickness; ST: sclera thickness; T-CPD: trabecular meshwork-ciliary process distance; I-CPD: iris-ciliary process distance; SD: standard deviation; ^*∗*^independent samples *t*-test.
